# The Legacy of Adversity? The Impact of Caregivers’ Childhood Experiences and Children’s Mental Health on Family Dynamics and Perceived Burden During the COVID-19 Pandemic

**DOI:** 10.3390/children12111519

**Published:** 2025-11-10

**Authors:** Julia Franziska Baschab, Kristina Feindel, Eva Moehler, Justine Hussong

**Affiliations:** Department of Child and Adolescent Psychiatry, Saarland University Hospital, 66421 Homburg, Germanyeva.moehler@uks.eu (E.M.); justine.hussong@uks.eu (J.H.)

**Keywords:** adverse childhood experiences (ACEs), child psychopathology, caregiver burden, family change, COVID-19 pandemic, intergenerational transmission, mental health

## Abstract

**Highlights:**

This study explores how caregivers’ adverse childhood experiences (ACEs) relate to their perceived COVID-19 burden, child psychopathology, and perceived changes in family relationships during the pandemic in a clinical sample of families.

**What are the main findings?**

- Higher caregiver ACEs were not directly linked to perceived COVID-19 burden but were indirectly linked via increased child psychopathology, which significantly predicted caregiver burden.
- While caregivers generally perceived pandemic-related family changes as slightly negative, higher ACEs were unexpectedly associated with less negative changes, especially in 2023.

**What are the implications of the main findings?**

- Child psychopathology plays a key role in the intergenerational transmission of stress, highlighting the need to address children’s mental health in families with high caregiver adversity.
- Unexpected resilience among high-ACE caregivers suggests that support systems, coping strategies, or shifted expectations may buffer family relationship burden during prolonged crises.

**Abstract:**

**Background:** This cross-sectional observational study examined the relationship between caregivers’ adverse childhood experiences (ACEs), their perceived COVID-19-related burden, child psychopathology, and changes in family relationships during the pandemic. **Methods:** The final sample included 285 children (*M* = 10.19, *SD* = 3.36) from clinical settings and their caregivers. Caregivers reported their own ACEs and their children’s psychopathology. Perceived caregiver COVID-19 burden and changes in family relationships were also assessed. Correlational analyses, regressions, and mediation models were conducted to test direct and indirect associations. **Results:** Caregivers reported an average of 1.63 ACEs, with 18.4% reporting four or more. Children of caregivers who reported four or more ACEs exhibited significantly elevated psychopathology scores. However, caregiver ACEs did not directly predict the perceived COVID-19 burden. A mediation analysis revealed that child psychopathology mediated the association between caregiver ACEs and caregiver COVID-19 burden. Unexpectedly, higher caregiver ACEs were associated with less negative perceived changes in family relationships, particularly in 2023, indicating heterogeneous family adjustment trajectories. **Conclusions:** These findings highlight that child psychopathology is a key mechanism in the intergenerational transmission of caregiver burden linked to childhood adversity. They also suggest that support systems, resilience, or differing expectations among high-ACE caregivers may help buffer changes in family relationship. Early identification and trauma-informed, family-centered interventions beyond acute crisis are essential. However, limitations include the reliance on caregiver self-report and cross-sectional design. Further, longitudinal, multi-informant research is needed to clarify these dynamics and inform targeted support strategies.

## 1. Introduction

The outbreak of the Severe Acute Respiratory Syndrome Coronavirus type 2 (SARS-CoV-2) marked the beginning of a global public health crisis with far-reaching and long-lasting consequences [1,2]. In response to the pandemic, governments worldwide implemented extensive containment measures, which fundamentally disrupted daily routines, social interactions and support structure [3]. Although the acute health threat has subsided, the psychological, social, and economic impacts of the Coronavirus disease 2019 (COVID-19) pandemic continue to shape individual well-being and family dynamics [4].

These effects have been particularly pronounced in vulnerable populations, such as children, families, and caregivers [5]. Caregivers of children with mental health disorders faced unique, multifaceted stressors during the pandemic. In Germany, repeated infection waves, nationwide lockdowns, and school closures disrupted routines, leading to limited access to healthcare and support systems which in turn exacerbated psychological distress [6]. These stressors not only intensified caregiver burden but also strained family dynamics, often resulting in heightened household conflict and reduced relationship quality.

However, not all caregivers were equally affected. Those with a history of adverse childhood experiences (ACEs) appear particularly vulnerable to psychological distress and difficulties in maintaining functional family relationships during crises, such as the COVID-19 pandemic [7]. ACEs refer to potentially traumatic experiences occurring before the age of 18, including various forms of abuse, neglect, and household dysfunction [8]. A meta-analysis by Hughes et al. [9] demonstrated the cumulative negative effects of ACEs on adult physical and mental health, stress regulation, and interpersonal functioning.

In Germany, Witt et al. [10] conducted a nationally representative study on ACEs, surveying over 2500 individuals aged 14 and older. At least one ACE was reported by 43.7% of participants, while 8.9% reported four or more. The most common experiences were parental separation (19.4%), substance abuse within the household (16.7%), emotional neglect (13.4%), and emotional abuse (12.5%). Individuals with four or more ACEs showed a significantly higher risk of depressive symptoms (OR = 7.8), anxiety (OR = 7.1), physical aggression (OR = 10.5), and reduced life satisfaction (OR = 5.1). These findings highlight the long-term psychological vulnerability associated with cumulative childhood adversity and underscore the importance of exploring how such experiences influence caregivers’ stress responses in times of crisis.

Previous research also points to the intergenerational consequences of ACEs. Children whose parents, particularly mothers, have experienced four or more ACEs are at a significantly increased risk of behavioral problems [11,12,13]. Parental emotional stress, often rooted in unresolved trauma, has been linked to reduced resilience and impaired parenting capacity [14]. Moreover, each additional maternal ACE has been associated with an 18% higher risk of developmental delays in children, especially in domains such as language, motor skills, social behavior, and problem-solving [15].

The longitudinal COPSY (Corona and Psyche) study conducted in Germany examined the psychosocial impact of the COVID-19 pandemic on children and adolescents [5]. Approximately 71% of children reported feeling burdened by contact restrictions, 65% found school and learning more exhausting, and over 37% of parents indicated more frequent conflicts with their children. Psychosomatic and psychological symptoms, including irritability, low mood, sleep disturbances, and physiological complaints such as headaches and stomach aches, became more prevalent. Families with additional risk factors, such as low parental education, migration background, or cramped living conditions, reported greater stress and reduced quality of life.

These findings are consistent with international research. In the United States, parental mental health has declined, and additional life stressors such as job loss and financial difficulties have become common [16,17]. In Italy, parental psychological distress, low resilience, and social isolation predicted increased caregiver burden [18]. Recent research highlights that parental childhood adversity and resilience jointly shape family functioning, parenting stress, and child outcomes during crises [19,20,21,22,23]. These results suggest that caregivers’ ACEs may indirectly influence their psychological burden through effects on child functioning and family processes, while resilience factors may buffer these effects.

Despite this growing evidence, most existing studies focus on general population samples and acute stressors, whereas intergenerational risk-factors, such as caregiver ACEs, remain understudied, particularly in clinical child and adolescent populations. Addressing this gap is essential to better understand mechanisms of vulnerability and resilience in families facing multiple, overlapping risks.

### 1.1. The Present Study

The present study examined the psychosocial impact of the COVID-19 pandemic on caregivers of children and adolescents with mental health disorders, focusing on caregiver burden, child psychopathology, and perceived changes in family relationships. Specifically, it investigated whether child psychopathology mediates the association between caregivers’ ACEs and their perceived COVID-19-related burden, reflecting intergenerational mechanisms of stress transmission. In addition, the study also explored patterns of perceived changes in family relationships, including potentially unexpected trajectories among caregivers with high ACEs. By focusing on a clinically referred population, this study addresses a key gap in the literature on intergenerational risk factors and resilience mechanisms in high-risk families.

### 1.2. Research Questions, Study Outline and Hypotheses

To address these objectives, this study used a prospective observational cohort design to collect clinical data and caregiver-reported questionnaires from 2021 to 2023. Participants were caregivers of children and adolescents receiving outpatient care at specialized clinics. Standardized questionnaires were used to assess caregiver burden related to the COVID-19 pandemic, perceived changes in family relationships, and caregivers’ history of adverse childhood experiences (ACEs), and child psychopathology. Based on this design, the study addressed the following research questions:

- Did caregivers of children with mental health disorders experience increased burden during the COVID-19 pandemic?
- How did caregivers perceive pandemic-related changes in family relationships?
- Are higher ACE scores among caregivers associated with increased caregiver burden and greater perceived changes in family relationships?
- Does child psychopathology mediate the association between caregivers’ ACEs and caregiver COVID-19-related burden? (exploratory)

Corresponding to these research questions, the following hypotheses were tested:

**Hypothesis** **1.a.** *There is a positive association between caregivers’ ACE scores and COVID-19 pandemic-related caregiver burden.*

**Hypothesis** **1.b.** *Caregiver burden related to the COVID-19 pandemic increased among caregivers of children with mental health disorders.*

**Hypothesis** **2.a.** *Caregivers perceive the COVID-19 pandemic-related changes in family relationships as negative.*

**Hypothesis** **2.b.** *There is a positive association between caregivers’ ACE scores and COVID-19 pandemic-related changes in family relationships.*

## 2. Materials and Methods

### 2.1. Sample

The present study was part of a larger research project consisting of four distinct sub-studies (see German Clinical Trial Register, DRKS00025986). Participants were recruited from the outpatient clinic of the Department of Child and Adolescent Psychiatry, University Medical Center Homburg and from a Child and Adolescent psychiatric and psychotherapeutic medical care center in Saarbrücken. Data collection was between August 2021 and December 2023. Inclusion criteria were an age between 0 and 17 years, informed consent to participate in the study, and sufficient German language skills of the caregiver. Written informed consent was obtained from all caregivers of participants, who completed the questionnaire on behalf of their child (1:1 correspondence). No formal sample size calculation was conducted due to the exploratory nature of this referred cohort study. The study was approved by the Ethics Committee of the Medical Association of Saarland (Ärztekammer des Saarlandes).

### 2.2. Study Design and Procedure

This study employed a repeated cross-sectional observational design in clinically referred patient cohorts, with caregivers recruited between 2021 and 2023. Each participant contributed data at a single time point, precluding causal or temporal inferences. Following informed consent, caregivers completed a standardized set of questionnaires, including the Child Behavior Checklist (CBCL), the Adverse Childhood Experiences (ACE) questionnaire, and a measure of COVID-19-related caregiver burden (COVID-19 burden). In addition, clinical diagnoses and descriptive data (gender and age) were extracted from patient records.

### 2.3. Measurements

Demographics. Sociodemographic data included the child’s age and gender as well as the month and year the questionnaire was completed.

Adverse childhood experiences (ACEs) of caregiver (ACE score caregiver). To assess the caregivers’ adverse childhood experiences, the German version of the Adverse Childhood Experiences Questionnaire (ACE-D) was used [8,24]. The ACE-D consists of 10 items covering three domains, abuse, household dysfunction, and neglect, based on the caregiver’s self-report. Completion takes approximately five minutes. The ACE-D has demonstrated good psychometric properties [25] and is widely used as a clinical screening tool.

Child psychopathology (CBCL score). For younger children (1.5–5 years), the CBCL/1½–5 [26] was used to assess psychological symptoms with 100 items. For older children and adolescents, the CBCL 6-18R [27] was administered, comprising 120 items across eight syndrome scales, which are grouped into internalizing, externalizing, and total problems categories. Average completion time is approximately 15 min.

COVID-19 burden of caregiver (COVID-19 burden). The COVID-19 Burden Questionnaire was administered to assess perceived pandemic-related stress and relationship changes [28]. It was developed by Döpfner [29] as part of the B-FAST study, a project evaluating the feasibility and acceptance of regular SARS-CoV-2 testing in German schools and childcare facilities. The caregiver-report questionnaire consists of 11 items assessing changes in relationships and perceived stress during the COVID-19 pandemic, rated on a 5-point scale ranging from −2 (much better) to +2 (much worse).

### 2.4. Data Analysis

All statistical analyses were performed using R Version 4.5.0 (R Core Team, Vienna, Austria) [30]. One-way ANOVAs were performed using the stats package, with effect sizes computed using effectsize Version 1.0.1 [31]. Lavaan Version 0.6-19 [32] was used for structural equation modeling and mediation analysis with bootstrapping. Regression models and post hoc regression tests were conducted with lmtest Version 0.9-40 [33] und emmeans Version 1.11.1 [34]. Missing values were handled by excluding participants with ≥4 missing items per questionnaire, for participants with fewer missing items, these values were ignored, and no imputation was performed. Model assumptions, including normality, multicollinearity and homoscedasticity were checked and met unless otherwise reported. Statistical significance was set at α = 0.05.

One-way ANOVAs with Tukey’s post hoc tests compared child psychopathology by caregiver ACE group (<4 vs. ≥4 ACEs). Two-proportion *z*-tests evaluated differences in ACE prevalence relative to a German reference sample. Pearson’s correlations examined associations among key variables.

Multiple linear regression models included predictors such as survey year contrasts, child age (*z*-standardized), gender, psychopathology scores (internalizing and externalizing, *z*-standardized), and caregiver ACE scores (*z*-standardized), with interaction terms to test moderation by survey year. Model fit and assumptions were checked prior to the analyses.

An exploratory mediation was tested via SEM with bootstrapped standard errors and confidence intervals (1000 resamples) to examine indirect effects of caregiver ACEs on caregiver COVID-19 burden via child psychopathology.

### 2.5. Bias and Limitations

Potential sources of bias include the reliance on caregiver self-report for both predictor and outcome measures. Complementary data sources, such as clinical diagnoses or professional reports, were extracted when available. This limitation is explicitly acknowledged and should be considered when interpreting the findings. Additionally, participants were recruited from clinical settings, which may limit generalizability to broader populations.

## 3. Results

### 3.1. Sample Characteristics

A total of 328 participants were initially recruited. The following were excluded from the final sample because of incomplete questionnaires (≥4 missing items): 14 participants who did not complete the ACE questionnaire; 11 participants who did not complete the CBCL questionnaire; 2 participants with four or more missing items on the ACE questionnaire; 1 participant due to missing sociodemographic information; 1 participant with four or more missing items on the COVID-19 burden scale; 7 participants who were mistakenly recruited after the data collection period. Thus, the final sample comprised N = 285 children aged between 3.6 and 17.7 years (*M* = 10.19, *SD* = 3.36; see Table 1). Although inclusion criteria allowed children aged 0–17 years, the youngest child in the final sample was 3.36 years, reflecting the actual patient population treated in the outpatient clinic and medical care center. The majority was male (62.1%).

### 3.2. Child Psychopathology

On average, children exhibited clinically relevant scores on the CBCL total scale (*M* = 65.12, *SD* = 11.29). A wide range of clinical diagnoses were reported, with multiple diagnoses per child being common. This reflects the high comorbidity in this clinical sample (see Appendix A, Table A1). The most frequent diagnosis was attention-deficit hyperactivity disorder (ADHD), predominantly hyperactive-impulsive type (F90.0), reported in 89 cases, followed by nonorganic enuresis (F98.0) in 81 cases, and nonorganic encopresis (F98.1) in 41 cases.

**Figure 1:**
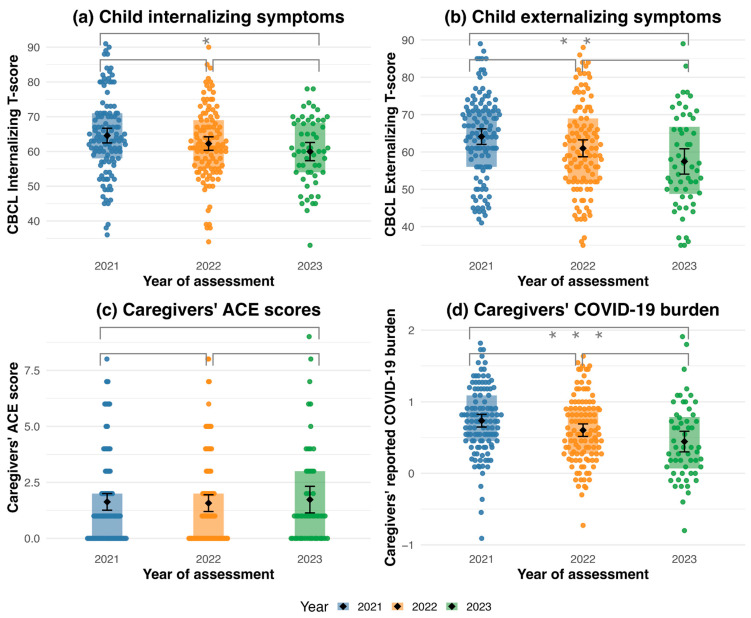
Children’s psychopathology internalizing (**a**) and externalizing (**b**), caregivers’ ACE (**c**) and caregivers’ perceived COVID-19 burden (**d**) from 2021 to 2023. Internalizing and externalizing scores from the CBCL. ACE = Adverse Childhood Experiences. COVID-19 burden = caregiver’s perceived pandemic-related burden, range: −2 (much better) to +2 (much worse). Boxes represent the interquartile range (25th–75th percentile), horizontal lines indicate the median, and dots reflect the individual data points. Diamonds indicate group means, with error bars representing the 95% CI. * *p* < 0.05, ** *p* < 0.01, and *** *p* < 0.001.

The means and standard deviations of internalizing scores of the CBCL by year (see Figure 1) were 2021 (*M* = 64.57, *SD* = 11.46), 2022 (*M* = 62.26, *SD* = 10.45) and 2023 (*M* = 59.96, *SD* = 9.81). A one-way ANOVA revealed a significant effect of year on internalizing scores, *F*(2, 282) = 3.66, *p* = 0.027, η^2^ = 0.03. Post hoc Tukey tests showed that internalizing scores in 2023 were significantly lower than in 2021 (b = −4.60, 95% CI [−8.73, −0.48], *p* = 0.024). Other pairwise year differences were not statistically significant (2021 vs. 2022: b = −2.31, 95% CI [−5.66, 1.04], *p* = 0.236; 2022 vs. 2023: b = −2.29, 95% CI [−6.43, 1.85], *p* = 0.394).

Means and standard deviations of externalizing scores of the CBCL by year were 2021 (*M* = 64.11, *SD* = 11.36), 2022 (*M* = 60.97, *SD* = 12.09) and 2023 (*M* = 57.46, *SD* = 12.70). A one-way ANOVA indicated a significant effect of year on externalizing scores in 2023, *F*(2, 282) = 6.10, *p* = 0.003, η^2^ = 0.04. Post hoc comparisons revealed that externalizing scores in 2023 were significantly lower in 2021 (b = −6.65, 95% CI [−11.22, −2.08], *p* = 0.002). Other pairwise year differences were not statistically significant (2021 vs. 2022: b = −3.14, 95% CI [−6.85, 0.57], *p* = 0.116; 2022 vs. 2023: b = −3.51, 95% CI [−8.10, 0.17], *p* = 0.171).

### 3.3. ACE of Caregivers

On average, caregivers who completed the questionnaire experienced 1.63 ACEs (*SD* = 2.05, range 0–9). Overall, 41.9% reported no ACEs, while 18.4% reported four or more ACEs (see Table 2). Parental divorce/separation (26.4%), mental illness in the household (25.9%) and emotional neglect (23.9%) were the most frequently reported ACEs.

Means and standard deviations of ACE scores by year were as follows (see Figure 1): 2021 (*M* = 1.63, *SD* = 2.02), 2022 (*M* = 1.58, *SD* = 2.01) and 2023 (*M* = 1.73, *SD* = 2.21). A one-way ANOVA showed no significant effect of year on ACE scores (*F*(2, 282) = 0.11, *p* = 0.896, η^2^ = 0.00). Post hoc pairwise comparisons using Tukey’s test revealed no significant differences between any years (all *p*_adj._ > 0.886).

### 3.4. Association Between Caregiver ACE and Child Psychopathology

To investigate whether children of caregivers with higher ACE exposure differ in symptom severity, a Welch Two Sample t-test was conducted comparing child psychopathology (CBCL total) between children of caregivers with less than four ACEs and those of caregivers with four or more ACEs. The results showed a significant difference, *t*(66.29) = −2.36, *p* = 0.021, indicating higher symptom scores for caregivers with four or more ACEs (M = 68.72, SD = 12.35) compared to caregivers with less than four ACEs (*M* = 64.26, *SD* = 10.62). The effect size was small to moderate (Cohen’s *d* = 0.41, 95% CI [0.10, 0.72, *r* = 0.16].

### 3.5. Comparison of ACE Scores with a German Reference Sample

To contextualize the prevalence of adverse childhood experiences (ACEs) of the caregivers in our sample, we compared our data with findings of a representative German population study by Witt et al. [10]. The reference sample included 2531 individuals aged 14 years and older (*M* = 48.6, *SD* = 18).

We conducted two-proportion z-tests to examine differences in the prevalence rates of individual ACE items and cumulative ACE categories between our sample and the reference sample (see Table 2). Results showed that several ACEs, including emotional abuse, physical abuse, sexual abuse, emotional neglect, physical neglect, parental separation/divorce, and household mental illness, were significantly more frequent in our sample (all *p*’s < 0.05). In contrast, rates of witnessed domestic violence, household substance use, and incarcerated household members did not differ significantly.

Regarding cumulative ACE burden, a significantly smaller proportion of caregivers in our sample reported zero ACEs compared to the reference sample (41.9% vs. 56.3%, *p* < 0.001). Furthermore, the proportion of individuals reporting four or more ACEs was significantly higher in our sample (18.4% vs. 8.9%, *p* < 0.001). These results suggest that our child psychiatric experienced a higher burden of caregiver childhood adversity than the general German population (see Table 2).

### 3.6. Perceived COVID-19 Burden of Caregivers

**Figure 2:**
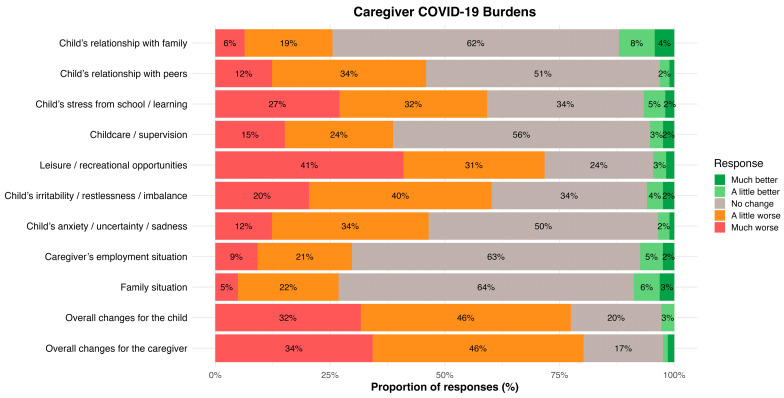
Distribution of caregivers’ perceived COVID-19 burden (2021–2023). *N* = 285. Caregivers reported a range of pandemic-related burdens affecting themselves and their children. Responses were measured on a scale ranging from −2 (much better) to 2 (much worse). Percentages are shown inside the bars. For visual clarity, fields without a number represent 1% of responses. Percentages may not sum to 100% due to rounding.

Caregivers reported a range of pandemic-related burdens affecting themselves and their children (see Figure 2). Based on the mean perceived COVID-19 burden score (ranging from −2 to +2), 8.1% of caregivers reported a reduction in burden (mean score < 0), 3.2% reported no change (mean score = 0), and 88.8% experienced an increase in burden (mean score > 0).

The means and standard deviations of perceived COVID-19 burden by year were as follows (see Figure 2): 2021 (*M* = 0.74, *SD* = 0.48), 2022 (*M* = 0.60, *SD* = 0.47) and 2023 (*M* = 0.44, *SD* = 0.54). An ANOVA revealed a significant effect of year on caregivers’ perceived COVID-19 burden, *F*(2, 282) = 6.93, *p* = 0.001, and η^2^ = 0.05, indicating differences in burden between 2021, 2022, and 2023. Post hoc comparisons using Tukey’s HSD test (see Figure 1) showed that caregivers reported significantly lower burden in 2023 than 2021 (b = −0.29, 95% CI [−0.48, −0.10], *p* < 0.001). The difference between 2021 and 2022 was not statistically significant (b = −0.13, 95% CI [−0.28, 0.02], *p* = 0.102), nor was the difference between 2022 and 2023 (b = −0.16, 95% CI [−0.35, 0.03], *p* = 0.116).

**Figure A1:**
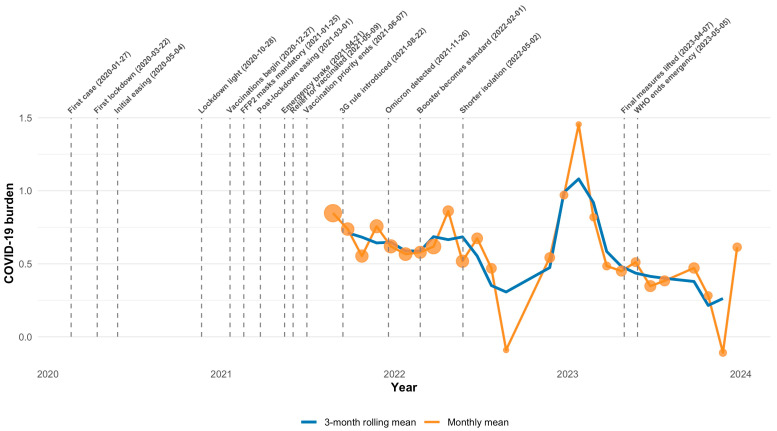
German COVID-19 timeline and COVID-19 burden of caregivers. The orange points represent the monthly mean values of the COVID-19 burden, with their size scaled proportionally to the number of participants (sample size) surveyed each month. The orange line connects these monthly means. The blue line shows the 3-month rolling mean, smoothing short-term fluctuations to highlight longer-term trends. Vertical dashed gray lines mark important COVID-19 milestone with corresponding labels above [55].

Figure A1 in Appendix B contextualizes the reported COVID-19 burden by displaying the timeline of the pandemic in Germany, including key events from 2020 to the end of 2023.

### 3.7. Predictor Selection Based on Correlational Analyses

To explore associations among the main study variables, bivariate Pearson correlations were computed. As shown in Table 3, significant correlations emerged between the outcome variable COVID-19 burden and several predictors. Specifically, higher levels of burden were associated with older child age, and elevated child psychopathology.

In all analyses involving child gender, one participant who identified as gender diverse was excluded because statistical comparisons were not feasible with a single case. This decision was made purely for statistical reasons and does not reflect any judgmental bias.

### 3.8. H1: Changes in Caregiver Burden Related to the COVID-19 Pandemic

A multiple linear regression was conducted to examine the association between caregivers’ ACE scores and perceived caregiver COVID-19 burden (Hypothesis 1a), as well as changes in perceived caregiver COVID-19 burden over the course of the pandemic among caregivers of children with mental health conditions (Hypothesis 1b). The dependent variable was COVID-19 burden (from −2 = much better to +2 much worse). The predictor variables included two contrasts for survey year (K1: 2021 vs. 2022/2023; K2: 2022 vs. 2023), child age (z-standardized), child gender (1 = male; −1 = female), child psychopathology internalizing (z-standardized) and externalizing (z-standardized) and parental ACE scores (z-standardized).

The overall model was significant (*F*(7, 276) = 8.41, *p* < 0.001), explaining 17.6% of the variance of caregiver burden (*R^2^* = 0.18, adjusted *R^2^* = 0.15), with a residual standard error of 0.46 (see Table 4).

Regarding Hypothesis 1a, ACE score was not a significant predictor of caregiver COVID-19 burden (b = 0.01, *p* = 0.815), providing no evidence of a direct association between caregiver ACEs and perceived caregiver burden.

For Hypothesis 1b, child internalizing (b = 0.09, *p* = 0.008) and externalizing symptoms (b = 0.10, *p* = 0.003) were significant positive predictors of caregiver burden. Higher levels of child psychopathology were associated with greater perceived worsening of caregiver burden during the pandemic.

The contrast comparing 2021 with 2022/2023 (K1) was marginally significant (b = 0.08, *p* = 0.060), suggesting that caregivers in 2021 reported slightly higher levels of perceived worsening of burden than caregivers in 2022 or 2023. However, the contrast when comparing 2022 and 2023 (K2) was not significant (b = 0.04, *p* = 0.288), indicating no substantial change in caregiver burden between these two years.

#### Exploratory Mediation Analysis of the Relationship Between Caregiver ACEs and Caregiver Burden via Child Psychopathology

**Figure 3:**
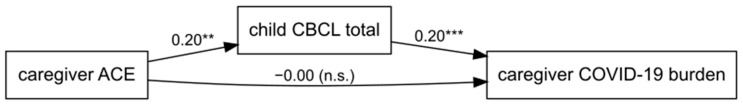
Mediation of the association between caregivers’ ACE score and COVID-19 burden via child CBCL total score. Estimates are reported. ** *p* < 0.01; *** *p* < 0.001.

A mediation analysis was conducted to examine whether children’s psychological symptoms (CBCL total scores) mediate the relationship between caregivers’ adverse childhood experiences (ACEs) and caregiver burden (see Figure 3). The model was tested using structural equation modeling (SEM) with bootstrapped standard errors and CIs (1000 resamples).

The SEM showed a saturated model with perfect fit indices, as expected given the just-identified structure (χ^2^ (0) = 0.00, CFI = 1.00, TLI = 1.00, SMR = 0.00). Caregiver ACEs significantly predicted child psychopathology (b = 0.20, *SE* = 0.07, *p* = 0.002, 95% CI [0.08, 0.33], which in turn significantly predicted caregiver burden (b = 0.20, *SE* = 0.03, *p* < 0.001, 95% CI [0.14, 0.25]. The direct effect of ACEs on caregiver burden was not significant (b = −0.00, *SE* = 0.04, *p* = 0.979, 95% CI [−0.08, 0.07]). However, the indirect effect of ACEs on caregiver burden via CBCL symptoms was significant (b = 0.04, *SE* = 0.01, *p* = 0.003, 95% CI [0.02, 0.07], indicating full mediation. These results suggest that the impact of caregivers’ ACEs on their perceived COVID-19 burden may be largely transmitted through their children’s increased psychological symptoms—as perceived by the caregivers themselves rather than via a direct effect.

### 3.9. H2: Changes in Family Relationships Related to the COVID-19 Pandemic

To assess whether caregivers perceived changes in their family situation due to the COVID-19 pandemic as negative, we used Item 9 (“Has your family situation changed as a result of the COVID-19 pandemic?”), rated on a scale from −2 (much better) to +2 (much worse). The item served as a single indicator of perceived changes in family relationships, directly reflecting the construct addressed in the hypotheses.

#### 3.9.1. H2.a: Average Perceived Changes in Family Relationships

A one-sample t-test was conducted to determine whether the mean rating of family relationship changes significantly from zero. Results showed a significantly positive mean (*M* = 0.20, *SD* = 0.75), indicating that, on average, caregivers generally perceived family relationships as slightly worse during the pandemic, *t*(281) = 4.43, *p* < 0.001, 95% CI [0.12, ∞], with a small effect size (Cohen’s *d* = 0.26).

#### 3.9.2. H2.b: Association Between Caregivers’ ACE Scores and COVID-19-Related Changes in Family Relationships

A hierarchical linear regression analysis examined the relationship between caregivers’ ACE and perceived family relationship changes. In step 1, predictors included survey year contrasts (K1: 2021 vs. 2022/2023; K2: 2022 vs. 2023), as well as internalizing and externalizing CBCL scores (z-standardized) and ACE score (*z*-standardized).

In step 2, interaction terms between the standardized ACE score and the two contrasts for survey year were added (see Table 5). This extended model was also statistically significant and showed improved fit, with *R^2^* = 0.06, adjusted *R^2^* = 0.03, *F*(7, 274) = 2.45, and *p* = 0.019.

The main effect of caregiver ACEs was significant, with b = −0.127, *p* = 0.007, indicating that higher caregiver ACEs were associated with less negative perceived family changes. The interaction terms (K1 x ACE and K2 x ACE) approached significance, indicating a trend-level moderation effect of survey year.

**Figure 4:**
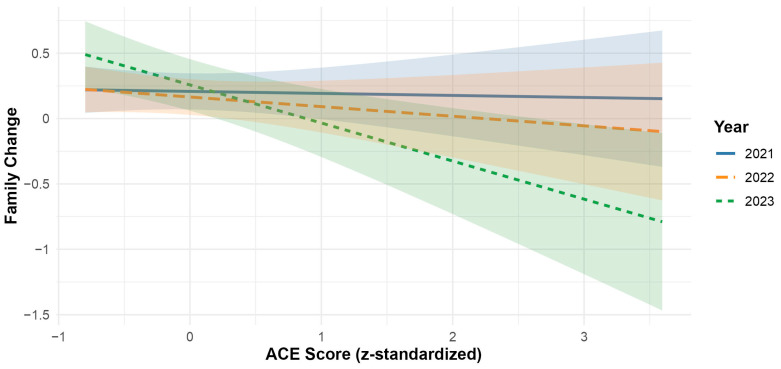
Interaction between survey year and caregivers’ ACEs on perceived family change. Predicted values of perceived changes in family relationships (CB_9r) are plotted as function of caregivers’ *z*-standardized ACE scores (ACE_score_z), with separate regression lines and 95% CI shown for each survey year: 2021 (solid line and blue), 2022 (dashed line and orange), 2023 (short-dashed line and orange). The model controls for *z*-standardized internalizing and externalizing symptoms. ACE = Adverse Childhood Experiences; Family Changes = coded from −2 (much better) to +2 (much worse).

Exploratory post hoc analyses revealed that the association between ACEs and perceived relationship changes was most pronounced in 2023 (b = −0.29, 95% CI [−0.48, −0.11], compared to 2021 (b = −0.02, 95% CI [−0.16, 0.12],) and 2022 (b = −0.07, 95% CI [−0.21, 0.07]). A Tukey-adjusted contrast showed that the 2023 estimate differed significantly from 2021 (b = 0.28, *SE* = 0.12, *t*(274) = 2.37 *p* = 0.049), while the other contrasts were not significant (2021 and 2022: b = 0.06, *SE* = 0.10, *p* = 0.831; and 2022 and 2023: b = 0.22, *SE* = 0.12, *p* = 0.152). This suggests that in 2023, caregivers with more ACEs perceived family relationships as significantly less negatively impacted compared to 2021 (see Figure 4).

To further complement the regression analysis, the Pearson correlation between ACE scores and perceived family relationship changes was calculated. The correlation was small, negative, but not statistically significant, with *r* = −0.10, *p* = 0.099, 95% CI [−0.21, 0.02], indicating a weak tendency for higher ACE scores to be associated with less negative perceptions of family changes.

## 4. Discussion

This study investigated how caregivers’ adverse childhood experiences (ACEs) relate to their perceived COVID-19-related burden and changes in family relationships, while accounting for the mental health of their children.

Our clinical sample included 285 child psychiatric patients (*M* = 10.19 years, *SD* = 3.36), with clinically elevated psychopathology scores (CBCL total score *M* = 65.12, *SD* = 11.29). On average, caregivers reported 1.63 ACEs, and 18.4% reported four or more. A higher number of caregiver ACEs was linked with greater child psychopathology, which in turn significantly predicted caregiver burden. While ACEs did not directly predict caregiver burden, child psychopathology mediated this association. Caregiver ACEs were also associated with less negatively perceived changes in family relationships, with this association being the strongest in 2023.

### 4.1. Caregiver ACEs and COVID-19 Burden (H1.a)

Contrary to the initial hypothesis, caregivers’ ACE scores were not directly associated with their perceived COVID-19-related burden. This finding contrasts with prior research suggesting that early adversity may sensitize individuals to later stressors [35,36,37]. One possible explanation could be that, during the early stages of the pandemic, external stressors (e.g., lockdowns, disruptions in care systems) were so persuasive that they may have overshadowed individual vulnerability factors such as childhood adversity. Another explanation, supported by the mediation analysis discussed below, is that ACEs affect perceived burden indirectly, through their influence on child psychopathology as perceived by the caregivers themselves.

### 4.2. Child Psychopathology and Caregiver Burden (H1.b)

As expected, higher levels of child internalizing and externalizing symptoms predicted greater caregiver burden. This aligns with prior research showing that child behavioral and emotional difficulties are a key predictor of parenting stress and burden [38,39,40]. The effect remained robust even after controlling for caregiver ACEs and survey year.

These findings also align with transactional models of development [41,42], which emphasize the bidirectional influence between children and their caregiving environment [43,44]. Elevated child psychopathology may increase caregiver burden, which may further exacerbate child difficulties, creating a vicious cycle. Such bidirectional dynamics may be especially pronounced during times of crisis, when families are exposed to additional stressors [5,45,46]. However, it needs to be taken into consideration that child psychopathology was assessed in caregiver report only and might thereby have been subject to perceptive distortion related to caregiver ACEs and not be an objective reflection of child behavior.

### 4.3. Exploratory Mediation Analysis

Although not part of our a priori hypotheses, an exploratory mediation analysis showed that child psychopathology significantly mediated the association between caregiver ACEs and perceived caregiver COVID-19-related burden. In line with intergenerational transmission models [47], caregivers with higher ACE scores reported greater child psychopathology, which was in turn linked to increased caregiver burden. This suggests that caregiver history may exert its effects on current functioning primarily through its impact on offspring mental health.

### 4.4. Perceived Changes in Family Relationships (H2.a and H2.b)

Consistent with previous research [5,7,48], caregivers generally perceived COVID-19-related changes in family relationships as slightly negative. Unexpectedly, as early life adversity is typically linked to poorer relational functioning and greater family stress during crises [7,9], higher caregiver ACE scores were associated with less negatively perceived changes in family relationships, particularly in 2023. Although the interaction between ACEs and survey year did not reach conventional significance thresholds, post hoc analyses suggested a trend-level moderation, with caregivers reporting comparatively stable or improved family relationships during the later phase of the pandemic. However, the small effect size suggests limited clinical relevance and should be interpreted with caution.

Several explanations are possible. Families with higher ACE exposure may have already been connected to support systems (e.g., mental health care, youth welfare services), which could have buffered negative impacts [49,50]. Also, they might have acquired coping strategies for dealing with adversity.

Furthermore, in 2021, when societal burden was collectively high (e.g., due to lockdowns and systemic disruptions), individual vulnerabilities may have been less visible, as nearly all caregivers experienced high stress levels. By 2023, as acute external stressors subsided, individual differences such as childhood adversity may have become more salient again. This delayed vulnerability effect may reflect the re-emergence of personal factors once short-term coping mechanisms fade or external support systems weaken during the post-crisis period.

Alternatively, caregivers with a history of adversity may initially function relatively well under crisis conditions by drawing on previously developed survival strategies [51,52]. It is possible that those caregivers initially coped well or even better than those without adversity during and after a crisis.

In addition, higher ACE exposure was also associated with greater child psychopathology in our sample. While this could be expected to increase family stress, the effect on perceived family relationships may be more complex. For instance, families already accustomed to managing high stress may have experienced fewer disruptions in relational dynamics or viewed their relationships more positively relative to past adversity.

Finally, we cannot rule out response biases. Given the overwhelmingly negative societal discourse surrounding the pandemic, caregivers without adversity might have reported relational changes as more negative in line with normative expectations. Conversely, caregivers with a history of adversity may have experienced any perceived stability or even minor improvement more positively, contrasting with their own childhood experiences or lower expectations. Importantly, caregiver ACE levels were stable across survey years, ruling out sampling effects as an explanation for the observed interaction. However, CBCL total scores did vary by year, with a peak in 2021, reflecting heightened child mental health challenges in caregiver report during the early pandemic phase. This is consistent with other studies reporting increased emotional and behavioral problems in children during this period [5,53,54].

### 4.5. Clinical Implications

Our findings have important implications for child and adolescent mental health services. Early screening for caregiver ACE exposure could help identify families at risk, particularly when child psychopathology is present. Family-centered, trauma-informed interventions may mitigate caregiver burden and intergenerational stress transmission. Furthermore, strengthening family resilience, through supportive communication, coping skills training, and access to resources, can buffer the impact of crises and support recovery. Post-pandemic, these interventions are crucial for vulnerable families, emphasizing both risk reduction and resilience promotion.

### 4.6. Limitations

Several limitations should be considered when interpreting the findings of this study.

First, the cross-sectional design precludes any conclusions about causality. Although mediation models were tested, the directionality of effects cannot be established. Longitudinal data are needed to confirm temporal pathways.

Second, all data were caregiver self-reports, which may introduce bias due to shared method variance. Caregivers’ own stress, adverse childhood experiences or emotional state could have influenced ratings of child psychopathology and family relationships, while societal narratives about pandemic hardship may have shaped responses. Nonetheless, the clinical nature of the sample and independently assessed child diagnoses increase the likelihood that reported symptoms reflect real impairments. Future research should employ multi-informant designs integrating child, clinician, and teacher perspectives.

Third, ACEs were assessed retrospectively and dichotomously, without capturing timing, duration, or subjective impact. These factors may moderate long-term effects and were not accounted for in the current analysis, limiting the depth of interpretation regarding adversity exposure.

Fourth, caregiver characteristics (e.g., age, mental health, socio-economic status, caregiving role) were not assessed. Such contextual factors may have influenced both perceived burden, child psychopathology and family dynamics. Caregiver mental health could represent a potential mediator between caregiver adversity, caregiver burden, and child psychopathology.

Fifth, the clinical nature of the sample, while ensuring relevance for high-risk populations, limits generalizability. Families in treatment may differ from community samples in stress exposure and resources. Thus, caution is warranted when extending these findings beyond clinical contexts.

### 4.7. Future Research

Building on the findings of this study, future studies should employ longitudinal, multi-informant, and mixed-method designs to disentangle causal pathways and reduce shared method variance. Qualitative approaches (e.g., caregiver or clinical interviews) could further illuminate how adverse experiences shape family perceptions and relational processes. Such approaches could help differentiate between caregiver perception and objective indicators of child functioning and family relationships.

More detailed assessments of caregiver adversity, including timing, duration, and subjective impact, would allow for a finer understanding of how specific ACE patterns affect family functioning. Moreover, it would be interesting to examine differences between directly and indirectly experienced ACEs. Expanding contextual variables (e.g., caregiver mental health, parenting practices, socio-economic status) could help identify risk and resilience mechanisms. Comparative studies across clinical community samples, diverse cultural contexts, are needed to test the generalizability of these findings and to address potential selection bias inherent to clinical samples.

Finally, intervention research should examine whether addressing caregiver trauma histories and enhancing family-level support can buffer intergenerational effects of early adversity, particularly during crises when structural supports are weakened and family systems are under heightened burdens.

## 5. Conclusions

This study contributes to the growing literature on the intergenerational transmission of adversity by demonstrating how caregivers’ adverse childhood experiences (ACEs) were associated with family functioning during the COVID-19 pandemic. In this clinical sample, while ACEs did not directly predict caregivers’ perceived burden, their impact was indirectly transmitted through perception of increased child psychopathology, underscoring the role of children’s mental health as a key pathway for the transmission of stress and burden within families. Unexpectedly, caregivers with more ACEs reported fewer negative changes in family relationships, particularly in 2023, suggesting trajectories shaped by both vulnerability and resilience.

These findings highlight the need for early identification and sustained support for vulnerable caregivers. Family-centered interventions can help interrupt intergenerational cycles of stress and foster resilience, particularly in high-risk populations. They may also inform secondary prevention strategies and provide targeted support before clinical deterioration occurs. Early screening for caregiver ACE exposure in child and adolescent mental health services is recommended, particularly in families with co-occurring child psychopathology, to enable tailored, trauma-informed interventions. Strengthening family resilience, through supportive communication, coping skills training, and access to resources, may buffer the impact of crises and promote recovery even among caregivers with adversity histories. Crucially, the full scope of the psychological and relational consequences of the COVID-19 pandemic remains unclear. The long-term impact, especially on already burdened families, is likely to unfold over the coming years. Without sustained research efforts, many of these delayed effects risk remaining invisible and unaddressed. It may take years to fully grasp the toll the pandemic has taken on child mental health and on vulnerable families. Ongoing longitudinal research is therefore essential to guide appropriate, evidence-based interventions and to ensure that those most affected are not overlooked as society moves forward.

## Figures and Tables

**Table 1:** Descriptive characteristics of sample.

Variable	*M* (*SD*)	Range	*N*	%
Age child	10.19 (3.36)	3.58–17.67	285	
Gender child			285	
male			177	62.1%
female			107	37.5%
diverse			1	0.4%
Year Questionnaire			285	
2021			116	40.7%
2022			113	39.6%
2023			56	19.6%
Psychopathology child				
CBCL Internalizing	62.75 (10.86)	33–91		
CBCL Externalizing	61.56 (12.14)	35–89		
CBCL Total Score	65.12 (11.29)	32–91		
Clinical diagnoses child ^a^			285	
Externalizing			151	53.0%
Internalizing			93	32.6%
Developmental			62	21.8%
Intellectual disability			2	0.7%
Other			241	84.6%
ACE score caregiver	1.63 (2.05)	0–9	285	
COVID-19 burden caregiver ^b^	0.63 (0.50)	−0.91–1.91	285	

Note. Percentages may not sum to 100 due to rounding. CBCL = Child Behavior Checklist 6-18R [27]; ACE = Adverse Childhood Experiences [8,24]. COVID-19 burden = questionnaire of Döpfner & Görtz-Dorten [28]. ^a^ Multiple diagnoses per child possible. ^b^ Responses from −2 (much better) to +2 (much worse).

**Table 2:** Comparison of ACE prevalence between study sample and the sample of Witt et al. [10].

	Study Sample (%)	Reference Sample (%)	95% CI	*p*-Value	Cohen’s *h*
ACE Frequencies					
Emotional abuse	23.3	12.5	[18.5, 28.7]	<0.001 ***	0.28
Physical abuse	16.5	9.1	[12.4, 21.4]	<0.001 ***	0.22
Sexual abuse	9.2	4.3	[6.1, 13.2]	<0.001 ***	0.20
Emotional neglect	23.9	13.4	[19.0, 29.2]	<0.001 ***	0.27
Physical neglect	7.4	4.3	[4.6, 11.0]	0.018 *	0.13
Parental separation/divorce	26.4	19.4	[21.4, 31.9]	0.004 **	0.17
Witnessed domestic violence	7.1	9.8	[4.4, 10.7]	<0.134	−0.10
Household substance use	19.9	16.7	[15.4, 25.0]	0.151	0.08
Mental illness in the household	25.9	10.6	[20.9, 31.4]	<0.001 ***	0.40
Incarcerated family member	4.2	3.5	[2.2, 7.3]	0.514	0.04
Cumulative ACE					
0 ACEs	41.9	56.3	[36.0, 48.0]	<0.001 ***	−0.29
1 ACEs	22.1	20.7	[17.3, 27.5]	0.600	0.03
2 ACEs	11.4	8.6	[7.9, 15.8]	0.104	0.09
3 ACEs	6.2	5.4	[3.7, 9.8]	0.502	0.03
≥4 ACEs	18.4	8.9	[14.0, 23.5]	<0.001 ***	0.28

Proportions refer to the percentage of participants (caregivers). Two proportion *z*-tests (two-tailed) were used for comparisons. Reference data are from Witt et al. [10]: a representative German population-based study (*N* = 2531; age ≥ 14 years, *M* = 48.6, *SD* = 18). * *p* < 0.05, ** *p* < 0.01, *** *p* < 0.001.

**Table 3:** Correlation matrix.

	Year	Child Age	Gender Child	CBCL Total	CBCL Ext.	CBCL Int.	COVID-19 Burden	ACE Care-Giver
Year	-							
Age Child	−0.32 ***	-						
Gender Child ^a^	−0.08	−0.05	-					
CBCL total ^b^	−0.21 ***	0.25 ***	0.00	-				
CBCL ext. ^b^	−0.21 ***	0.08	0.05	0.87 ***	-			
CBCL int. ^b^	−0.16 **	0.31 ***	−0.11	0.79 ***	0.52 ***	-		
COVID-19 burden	−0.22 ***	0.20 ***	0.06	0.39 ***	0.33 ***	0.33 ***	-	
ACE caregiver ^c^	−0.02	−0.02	0.02	0.18 **	0.18 **	0.13 *	0.11	-

Note. Pearson correlation coefficients are reported. Although Year is a categorial variable with three levels (2021, 2022, 2023), it was treated as numeric to assess linear trends over time. Interpretations should be limited to linear trends across years. ^a^ Child gender: 1 = male, −1 = female. ^b^ CBCL = Child behavior Checklist (sc. = score; ext. = externalizing; int. = internalizing), T-scores. ^c^ ACEs = Adverse Childhood Experiences of caregivers. * *p* < 0.05; ** *p* < 0.01; *** *p* < 0.001.

**Table 4:** Linear regression results predicting caregiver COVID-19 burden (H1).

Predictor	Estimate	Std. Coeff.	95% CI	*t*-Value	*p*-Value
K1 ^a^	0.08	0.11	[0.00, 0.23]	1.89	0.060
K2 ^b^	0.04	0.06	[−0.05, 0.18]	1.06	0.288
Age child (*z*)	0.04	0.09	[−0.03, 0.21]	1.43	0.154
Gender ^c^	0.03	0.06	[0.04, 0.17]	1.63	0.246
CBCL Int. (*z*)	0.09	0.18	[0.05, 0.32]	2.67	0.008 **
CBCL Ext. (*z*)	0.10	0.20	[0.07, 0.33]	3.05	0.003 **
ACE score (*z*)	0.01	0.01	[−0.10, 0.12]	0.23	0.815

Note. N = 284. Residual df = 276. ^a^ K1 = 2021 vs. 2022 and 2023. ^b^ K2 = 2022 vs. 2023. ^c^ gender coded as male = 1; female = −1. CBCL (T-scores). Age child and ACE were *z*-standardized. Standardized coefficients and 95% CIs were computed from standardized variables; unstandardized estimates, *t*-values and *p*-values are from the original model. Model assumptions were checked: Residuals approximated normality, homoscedasticity was confirmed, no auto correlation was detected, multicollinearity was low, and no influential outliers were found. ** *p* < 0.01.

**Table 5:** Linear regression results predicting caregivers’ perceived family changes (H2.b).

Predictor	Estimate	Std. Coeff.	95% CI	*t*-Value	*p*-Value
K1 ^a^	−0.00	0.00	[−0.12, 0.12]	−0.04	0.972
K2 ^b^	−0.05	−0.05	[−0.17, 0.07]	−0.76	0.448
Age child (*z*)	−0.13	−0.12	[−0.24, −0.01]	−2.73	0.007 **
Gender ^c^	0.08	0.10	[−0.03, 0.24]	1.51	0.131
CBCL Int. (*z*)	0.08	0.10	[−0.03, 0.24]	1.50	0.136
CBCL Ext. (*z*)	0.11	0.11	[−0.01, 0.23]	1.83	0.069
ACE score (*z*)	0.10	0.11	[−0.01, 0.22]	1.86	0.064

Note. N = 284. Residual df = 276. ^a^ K1: 2021 vs. 2022 and 2023. ^b^ K2: 2022 vs. 2023. ^c^ refers to Child gender (1 = male, −1 = female). Age child, CBCL (T-scores) and ACE were *z*-standardized. Standardized coefficients and 95% CIs were computed from standardized variables; unstandardized estimates, *t*-values and *p*-values are from the original model. Model assumptions were checked: Residuals approximated normality, homoscedasticity was confirmed, no auto correlation was detected, multicollinearity was low, and no influential outliers were found. ** *p* < 0.01.

## Data Availability

The data presented in this study are available on request from the corresponding author due to sensitive nature of clinical data and the need to protect participant confidentiality.

## Abbreviations

The following abbreviations are used in this manuscript:

ANOVA	Analysis of Variance
CFI	Comparative Fit Index
CI	Confidence Interval
OR	Odds Ratio
TLI	Tucker–Lewis Index
SRMR	Standardized Root Mean Square Residual

## Appendix A

**Table A1:** Clinical diagnoses of children (total *N* = 285).

Group		Diagnosis	*N*
Externalizing			151
	ADHD	Disturbance of activity and attention (F90.0; 89); Hyperkinetic conduct disorder (F90.1; 13)	102
	ODD	Oppositional defiant disorder (F91.3; 35)	35
	Conduct Disorder	Conduct disorder confined to the family context (F91.0; 3); Conduct disorder with lack of social bonds (F91.1; 1); Depressive conduct disorder (F92.0; 4); Other mixed disorders of conduct and emotions (F92.8; 5); Unspecified mixed disorder of conduct and emotions (F92.9; 1)	14
Internalizing			93
	Depressive disorders	Mild depressive episode (F32.0; 11); Moderate depressive episode (F32.1; 16); Severe depressive episode with psychotic symptoms (F32.3; 1); Prolonged depressive reaction (F43.21; 1)	29
	Anxiety disorders	Agoraphobia with panic disorder (F40.01; 1); Panic disorder (F41.0; 5); Generalized anxiety disorder (F41.1; 1); Social phobias (F40.1; 11); Specific phobias (F40.2; 4)	22
	PTSD & Adjustment	Post-traumatic stress disorder (F43.1; 7); Adjustment disorders (F43.2; 12); Reaction to severe stress, unspecified (F43.9; 1)	20
	Child-hood anxiety	Social anxiety disorder of childhood (F93.2; 3); Separation anxiety disorder of childhood (F93.1; 6); Phobic anxiety disorder of childhood (F93.0; 1)	10
	Other	Other childhood emotional disorders (F93.8; 7)	7
	OCD	Obsessive–compulsive disorder with predominant obsessions (F42.0; 1); Mixed obsessional thoughts and acts (F42.2; 4)	5
Developmental			62
	Scholastic skills	Specific reading disorder (F81.0; 13); Specific spelling disorder (F81.1; 7); Specific disorder of arithmetical skills (F81.2; 4); Mixed disorder of scholastic skills (F81.3; 1); Developmental learning disorder, unspecified (F81.9; 1)	26
	Mixed	Mixed specific developmental disorders (F83; 15)	15
	Speech	Phonological disorder (F80.0; 4); Expressive language disorder (F80.1; 3); Receptive language disorder (F80.2; 2)	9
	Autism spectrum	Childhood autism (F84.0; 1); Atypical autism (F84.1; 2); Asperger’s syndrome (F84.5; 5); Pervasive developmental disorder, unspecified (F84.9; 1)	9
	Motor	Specific developmental disorder of motor function (F82; 1); Developmental disorder of fine and graphomotor coordination (F82.1; 1); Developmental motor disorder, unspecified (F82.9; 1)	3
Intellectual disability	Mild- Unspecified	Mild intellectual disability with/without behavioral impairment (F70; 1); Unspecified mental retardation with significant behavioral problems (F79.1; 1)	2
Other			241
	Incontinence	Nonorganic enuresis (F98.0; 81); Nonorganic encopresis (F98.1; 41); Other specified urinary incontinence (R32.8; 37); Reflex incontinence (R32.0; 8)	167
	Behavioral and emotional disorders	Other specified behavioral and emotional disorders with onset usually occurring in childhood and adolescence (F98.8; 19); Elective mutism (F94.0; 4); Disinhibited attachment disorder of childhood (F94.2; 2); Stuttering (Stammering; F98.5; 1)	26
	Tic Disorder	Transient tic disorder (F95.0; 4); Chronic motor or vocal tic disorder (F95.1; 3); Tourette syndrome (F95.2; 1)	8
	Eating Disorders	Other eating disorders (F50.8; 3); Atypical bulimia nervosa (F50.3; 2); Anorexia nervosa (F50.0; 2); Atypical anorexia nervosa (F50.1; 1)	8
	Gender Identity Disorders	Transsexualism (F64.0; 1); Gender identity disorder of childhood (F64.2; 1); Other gender identity disorders (F64.8; 1)	3
	Dissociative & Psychotic Disorders	Dissociative motor disorders (F44.4; 1); Disorganized schizophrenia (F20.1; 1)	
	Sleep Disorders	Nonorganic insomnia (F51.0; 2)	2
	Impulse Control Disorders	Other specified habit and impulse disorders (F63.8; 2)	2

*Note.* Multiple diagnoses per child were possible. Diagnoses are based on ICD-10 codes. The numbers in parentheses after each diagnosis refer to the number of children diagnosed with that specific condition.
